# Usability testing of EatsUp®: mobile application for monitoring balanced dietary practices and active lifestyle among adolescents—a study in Jakarta, Indonesia

**DOI:** 10.3389/fdgth.2025.1506952

**Published:** 2025-06-13

**Authors:** Erfi Prafiantini, Rina Agustina, Betty Purwandari, Dian Novita Chandra, Dini Rahma Bintari, Fellatinnisa Zafira Rajwadini, Jihan Farhanah, Aryono Hendarto

**Affiliations:** ^1^ Department of Nutrition, Faculty of Medicine, Universitas Indonesia—Dr. Cipto Mangunkusumo General Hospital, Jakarta, Indonesia; ^2^ Human Nutrition Research Center, Indonesian Medical Education and Research Institute (HNRC IMERI), Faculty of Medicine, Universitas Indonesia, Jakarta, Indonesia; ^3^ Department of General Practitioner, Universitas Indonesia Hospital, Universitas Indonesia, Depok, West Java, Indonesia; ^4^ Faculty of Computer Science, Universitas Indonesia, Depok, Indonesia; ^5^ Faculty of Psychology, Universitas Indonesia, Depok, Indonesia; ^6^ Department of Child Health, Faculty of Medicine, Universitas Indonesia—Dr. Cipto Mangunkusumo General Hospital, Jakarta, Indonesia

**Keywords:** usability testing, user experience, mobile application, balanced diet, active lifestyle, adolescents

## Abstract

**Background:**

Adolescence is a critical period for establishing lifelong health habits, yet many adopt unhealthy behaviors, leading to obesity and other non-communicable diseases. Mobile apps offer a promising platform for delivering health interventions through education. Usability testing is essential to ensure mobile app features align with adolescent preferences and promote sustained behavior change.

**Methods:**

We conducted an experimental usability study from June to August 2024 in Jakarta, Indonesia targeting adolescents aged 15–18 who used the EatsUp® mobile application. Participants engaged with the app for seven consecutive days, completing daily tasks and a user experience questionnaire. User experience was assessed across six domains—Attractiveness, Perspicuity, Efficiency, Dependability, Stimulation, and Novelty—using a 7-point Likert scale. Descriptive statistics were used to analyze the data, which were compared against established user experience benchmarks.

**Results:**

A total of 30 high school students participated (mean ± SD age 16 ± 0.70 years), of whom 23 (76.7%) were female. Most participants (90.0%) used the EatsUp® application for at least seven consecutive days. The app received positive and high user experience ratings across all six parameters, with mean scores exceeding 0.8. Compared to the benchmark data from previous UEQ studies, the app ranked in the “Excellent” category (top 10%) for five parameters, while Perspicuity was rated as “Good” (top 25%).

**Conclusion:**

The *EatsUp*® app demonstrated strong usability, with an overall positive user experience. It ranked as “Excellent” in five user experience parameters except perspicuity, making it well-suited for adolescents. However, perspicuity needs improvement to enhance ease of use. Study limitations include a predominantly female sample from Jakarta-based schools, limiting generalizability. Future studies should include a more diverse population and explore features like gamification to enhance long-term engagement.

## 1 Introduction

Adolescence is a crucial period for developing behaviors related to nutrition and health, as habits formed during this stage often persist into adulthood. During this time, they have transformative growth and openness to change, presenting unique opportunities to adopt dietary and physical activity modification that can provide a foundation for a healthy lifestyle in later life (1). Unfortunately, a growing number of adolescents are leading unhealthy lifestyles that include eating poorly, being less active, and spending a lot of time in front of screens. These behaviors are causing obesity and leading to non-communicable diseases, particularly type 2 diabetes mellitus, and cardiovascular problems, which are increasingly prevalent around the world (2). Previous studies showed that nowadays, adolescents in Indonesia tend to skip breakfast and overindulge in unhealthy snacks. They often buy processed or junk food from food vendors at school instead of nutritious home-cooked meals (3). Adolescents also engage more with smartphones, contributing to physical inactivity and obesity (4). In Indonesia, there has been a significant increase in adolescent obesity. According to the Indonesian National Health Survey (SKI), between 2013 and 2023, the prevalence of obesity in children aged 13–15 years increased from 2.5% to 4.1%, while in those aged 16–18 years increased from 1.7% to 3.3% (5). These figures highlight the growing health risk faced by Indonesian adolescents and underscore the urgency of early an effective interventions. Therefore, combining physical activity and dietary changes through nutrition, food provision, and lifestyle education can be the best way to address adolescent obesity and prevent them from developing non-communicable diseases in later life (2, 6).

The digital habits of adolescents further support the need for innovative and technology-based interventions. Data from the 2023 Indonesian Central Bureau of Statistics (BPS) indicated that 92.1% of smartphone users were dominated by individuals aged 15–24 years (7). Additionally, a study by the Indonesian Internet Service Providers Association (APJII) in 2024 reported that 90.3% of internet users aged 13–18 years accessed the internet using smartphones (8). As adolescents are more likely to use smartphones, traditional health promotion methods may fail to engage this tech-savvy demographic effectively. Mobile health apps are increasingly utilized as digital interventions across various healthcare sectors (9). They offer a powerful platform for delivering health interventions targeted to adolescents. A study demonstrated that mobile apps can increase adherence to self-monitoring in intervention programs (10). Mobile apps can also help deliver health-related content that encourages healthy lifestyle behaviors. It can help effectively and practically educate and motivate adolescents about their health by providing educational messages, audio, and video files (11). Additionally, the apps may help users track their physical activity, nutrition intake, and physiological well-being (12).

However, mobile health apps for adolescents need to consider their digital habits and interests. Adolescents are usually drawn to interactive, gamified features and real-time feedback, which help sustain engagement and motivate consistent use (11, 12). Because of this, usability is a top priority while developing mHealth apps. A study showed that a mobile app's usability testing helps refine the development of an adolescent obesity management app (13). Usability testing is essential in ensuring the app is accessible and user-friendly since it assesses how well users can interact with it (14). For mobile health apps targeting adolescents, the developer should incorporate feedback from representative users throughout the development process to shape features, user interface design, user experience, and overall technical effectiveness so that functionality can be optimized (13). Developers can also find potential engagement obstacles, including complicated user flows or confusing instructions, and fix them before the app is released to the public by including representative users in the usability testing process. Without this step, even the most well-designed mobile health can fail to achieve its objectives due to user disengagement or frustration with its functionality (15). By aligning the app's design and functionality with the target user's expectations, developers can create mHealth apps that deliver health education and engage users in sustained and meaningful behavior change.

One widely used and well-regarded standardized tool for evaluating usability is the User Experience Questionnaire (UEQ). In Indonesia, UEQ is the most frequently used questionnaire in usability evaluation (16). The UEQ is a standardized survey instrument designed to assess the subjective user experience of an app, covering six dimensions: (1). Attractiveness, (2). Perspicuity, (3). Efficiency, (4). Dependability, (5). Stimulation, and (6). Novelty. These dimensions enable researchers and developers to gain insights into users' subjective responses, as well as the overall usability of an app. With these six dimensions, it is possible to determine areas of improvement (17). Attractiveness is a dimension of pure valence. On the other hand, dependability, efficiency, and precision are pragmatic or ergonomic qualities (goal-oriented). These dimensions provide developers with information regarding the app's efficiency and efficacy. The stimulation and novelty are hedonic qualities (not goal-oriented); with these dimensions, developers can learn about their users' preferences for app interface design (18). The UEQ is a reliable and valid measurement for user experience. It has already been validated through 11 usability tests with 144 participants and an online survey with 722 participants. It demonstrates high reliability, with each parameter exceeding the Cronbach's Alpha threshold of 0.7 consistency (measured by Cronbach's alpha) and good construct validity (18, 19). The UEQ has been validated for evaluating user experience in mobile app, making it a suitable instrument for developers and academic researchers to assess the usability of mobile apps (20). The UEQ is also easy for users because the questionnaire is simple and has an Indonesian version. Additionally, UEQ provided a tool to help researchers or developers analyze the usability testing results (17, 18).

EatsUp® is a mobile health app designed to promote healthy dietary habits. The app allowed users to input their weight. height and date of birth, which are then automatically used to calculate their BMI, dietary needs, and nutrition status classification. Additionally, the users can track their dietary intake by entering specific types of foods or beverages along with their respective nutritional data. This information is then used to build a detailed dietary record. Furthermore, the app provides access to nutrition education, empowering users to learn more about healthy eating. The previous version of EatsUp® used dietary needs calculation, nutrition status classification, and nutrition education contents designed for adults, whereas the current version has been adapted by replacing these three features to be more specific for adolescents. Prior research has evaluated the app's acceptance of EatsUp®. However, its usability has not yet been formally evaluated (21). Since usability is crucial in user engagement and long-term adoption, a comprehensive usability assessment is essential to ensure the apps align with adolescent preferences and support sustained healthy eating behaviors. This study addresses this gap by examining the role of mobile apps in promoting healthy lifestyles among adolescents, emphasizing the importance of usability testing in the app development process. Specifically, it aims to evaluate EatsUp® user experience, assessing its effectiveness, efficiency, and user satisfaction using the UEQ to determine whether it aligns with adolescents' preferences.

## 2 Methods

### 2.1 Ethical considerations

This study is part of the research titled “Development of the Mindful Eating Intervention (MEI) Model with the EatsUp® Application and the Impact of MEI on Body Composition, Insulin Resistance, and Diet Quality in Obese Adolescents” and received ethical approval from the Health Research Ethics Committee of the Faculty of Medicine, Universitas Indonesia—Dr. Cipto Mangunkusumo General Hospital (FKUI—RSCM) under ethical clearance number KET-1444/UN2.F1/ETIK/PPM.00.02/2023. Participants were provided with a comprehensive participant information sheet in the informed consent/assent documents, and all participants, along with their parents, provided an electronic signature and checked the consent box before participating in the study.

### 2.2 Eatsup® mobile application

This study utilized the EatsUp® application. The app was previously used in a study on obesity management in adults, and it successfully increased adherence to obesity management programs. The app was developed by collaborating with information technology application developers, graphic designers, communication experts, physicians, and dietitians. The EatsUp® core features included estimated caloric needs, dietary and physical activity monitors, simple menu recommendations, health news, notifications, a food database, estimated portion size, and pictures (21). Since EatsUp® successfully increased adherence to obesity management programs in adults, the application was selected for further development and adaptation to meet the needs of adolescents, particularly in promoting balanced diets and physical activity as it was planned to be used as intervention tools in the main research which applied balanced diet, physical activity, and mindful eating for treating obese adolescents. Because of the differences in the subject of the previous study, the research team and developer made several modifications to the app's features to better align with adolescents' needs and preferences.

**Figure 1:**
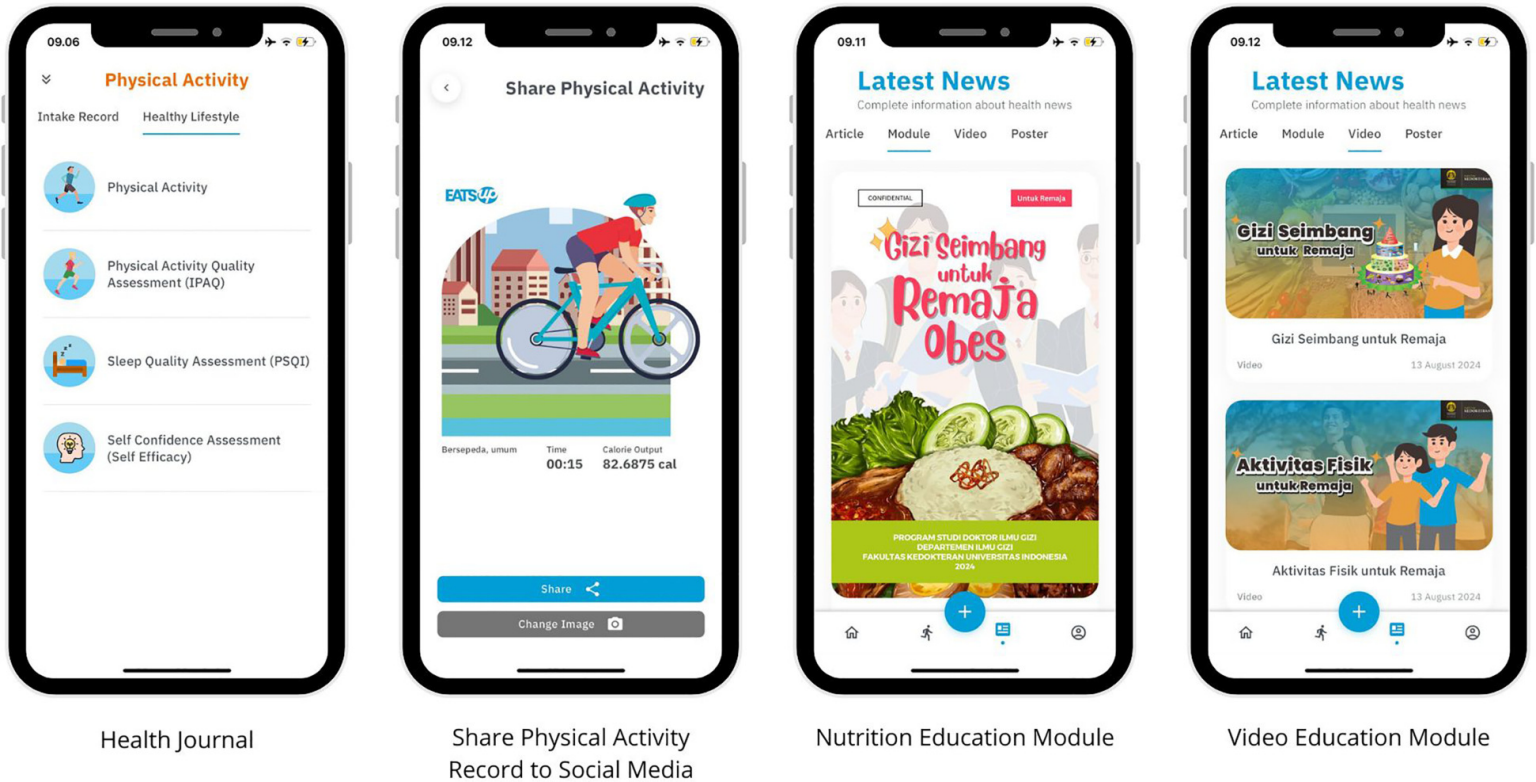
Screenshots of EatsUp® key features. Screenshots from EatsUp app, Fakultas Kedokteran Universitas Indonesia.

One of the features altered in this study was the Basal Metabolic Rate (BMR) calculator. The BMR calculator was originally created for adults. However, it was modified to calculate BMR specifically for adolescents using the Recommended Dietary Allowances (RDA) formula for children and adolescents, enabling users to view their daily caloric needs based on their respective ideal body weight. In addition, to allow adolescents to identify their nutritional status, the BMI-for-age calculation feature was adapted referring to the CDC BMI-for-age grow charts for ages 2–20 years. Additional features include Lock-Unlock User System for admins, allowing researchers to control user data when the app is used as a tool in nutrition interventions. Other features include social media sharing, enabling adolescents to share their physical activity records, developer account verification, and availability for the Android and iOS platforms (see Figure 1).

### 2.3 Study design and data collection

This research is an experimental study where usability testing was conducted using quantitative data collection from June to August 2024 through the User Experience Questionnaire (UEQ), an established standardized UX questionnaire used to measure user experience based on six parameters: attractiveness, efficiency, perspicuity, dependability, stimulation, and novelty of the EatsUp® Mobile Application in managing balanced diet and physical activity for adolescents (22).

The Usability Testing participants were high school students aged 15–18 years from Senior High School and Vocational High School in Central and East Jakarta who had used the EatsUp® application for at least 7 days. UEQ guidelines require a sample size of 20–30 respondents to achieve reliable results (19). Prior research on questionnaire-based usability testing indicates that a minimum of 12–14 participants is needed for reliable results (23). While other studies recommend at least 20 users for quantitative usability assessments (24). In this study, the UEQ was administered to 30 respondents selected using quota and purposive sampling based on the specified criteria. The age range of 15–18 years was chosen considering that at the age of 15, strong development of metacognitive understanding emerges, including knowledge, language development, and the ability to conduct decision-making. Thus, from age 15 onwards, adolescents achieve basic competencies to exercise their autonomy fully and to articulate their perspectives, particularly relating to their experience in using mobile apps (25). In addition, they have higher screen time when using digital media than younger groups below 13 (26).

Adolescent participants were approached and recruited through their schools based on recommendations from the Public Health Centre of the Special Region of Jakarta and via online posters distributed following prior research socialization. Students interested in participating filled out a registration link and completed informed consent before joining a WhatsApp group. Participants were then provided with a guide on how to use the EatsUp® application to record daily intake and physical activities. They were given daily challenge tasks, including downloading the EatsUp® application, creating an account, accessing food and physical activity tracking features, reading educational modules, and watching videos. After seven days of accessing the application, participants were invited to complete the UEQ via Google Forms. The links were distributed through WhatsApp groups to the enrolled adolescent participants. Data were collected within seven days after the end of the task to minimize recall bias. The questionnaires were kept short to prevent participants from feeling tired or losing focus while answering.

### 2.4 Outcome measures

The questionnaire distributed to high school adolescent students was divided into two main parts: participant characteristics and the User Experience Questionnaire (UEQ). The original German version of the UEQ was developed in 2005 using a data-driven approach to ensure practical relevance. It consisted of 26 question items from six parameters (17). This study utilized the UEQ, which has been translated into Indonesian using the translate-back translation method and validated in prior research (19, 27).

The first part of the questionnaire included questions about the sociodemographic characteristics of the participants, such as age, gender, school, domicile, and the duration of EatsUp® mobile application usage. The second part comprised the User Experience Questionnaire (UEQ), which consists of the following six parameters, with each item presented as a semantic differential on a seven-point scale ranging from 1 to 7, which then scored as −3 (most negative) to +3 (most positive) (Table 1).

**Table 1:** User experience (UX) parameters.

UX parameters	Question topics	Item and scoring
Attractiveness	User's overall impression of the product, whether they like it or not	−3 = Annoying +3 = Enjoyable
		−3 = Good +3 = Bad
		−3 = Unlikable +3 = Pleasing
		−3 = Unpleasant +3 = Pleasant
		−3 = Unattractive +3 = Attractive
		−3 = Unfriendly +3 = Friendly
Efficiency	User's perception of the product's efficiency and speed and the clarity of its interface	−3 = Slow +3 = Fast
		−3 = Inefficient +3 = Efficient
		−3 = Impractical +3 = Practical
		−3 = Cluttered +3 = Organized
Perspicuity	User's impression of the ease of understanding how to use the product	−3 = Not understandable +3 = Understandable
		−3 = Difficult to learn +3 = Easy to learn
		−3 = Complicated +3 = Easy
		−3 = Confusing +3 = Clear
Dependability	User's perception of the security and reliability of interacting with the product	−3 = Unpredictable +3 = Predictable
		−3 = Obstructie +3 = Supportive
		−3 = Not secure +3 = Secure
		−3 = Does not meet expectations +3 = Meet expectations
Stimulation	User's impression that using the product is engaging and enjoyable	−3 = Inferior +3 = Valuable
		−3 = Boring +3 = Exciting
		−3 = Not interesting +3 = Interesting
		−3 = Demotivating +3 = Motivating
Novelty	The impression that the product's design is innovative, creative, and attention-grabbing	−3 = Dull +3 = Creative
		−3 = Inventice +3 = Conventional
		−3 = Usual +3 = Leading edge
		−3 = Conservative +3 = Innovative

Perspicuity, Efficiency, and Dependability are categorized as pragmatic scales; Stimulation and Novelty are hedonic scales; and Attractiveness is considered a pure valence scale, reflecting the overall impression without linking it to specific interaction attributes between the user and the product. Each scale consists of four items presented as semantic differentials on a 7-point Likert scale, where each item pairs opposing terms representing a UX dimension, such as attractive-unattractive (28, 29).

### 2.5 Data analysis

The UEQ results were processed using Microsoft Excel tools, available in the UEQ Handbook. The data obtained from the UEQ questionnaire were analyzed to determine user satisfaction levels across the six parameters. The UX quality of the EatsUp® mobile application is then assessed by evaluating the mean scores and presented in a benchmark graph. The standard interpretation of scale means is as follows: mean scores between −0.8 and 0.8 indicate a neutral evaluation of the respective scale, and scores greater than 0.8 indicate a positive user evaluation. In contrast, scores below −0.8 reflect a negative evaluation (19). The measured scale means are compared against existing values from a benchmark data set compiled by previous UEQ researchers. Benchmark data was created to enable researchers to interpret the results of the UEQ compared to previous UEQ studies. This benchmark was developed by collecting data from all available UEQ evaluations from many researchers who shared the results of their UEQ evaluation studies (28). This benchmark data set includes data from 21,175 participants across 468 studies on various products, updated annually. The mean and standard deviation from the benchmark data set were calculated, which were then used as cutoff points to assess the UEQ scores of a given product (19, 30) (Table 2).

**Table 2:** Benchmark scale means interval for the UEQ parameters.

Parameter	Excellent	Good	Above average	Below average	Bad
Attractiveness	≥1.84	1.58–1.83	1.18–1.57	0.69–1.17	<0.69
Efficiency	≥1.88	1.50–1.87	1.05–1.49	0.60–1.04	<0.60
Perspicuity	≥2.00	1.73–1.99	1.20–1.72	0.72–1.19	<0.72
Dependability	≥1.70	1.48–1.69	1.14–1.47	0.78–1.13	<0.78
Stimulation	≥1.70	1.35–1.69	1.00–1.34	0.50–0.99	<0.50
Novelty	≥1.60	1.12–1.59	0.70–1.11	0.16–0.69	<0.16

The interpretation of the rating categories for a mean of each parameter is as follows (29):

• Excellent: The evaluated product is among the top 10% of results.
• Good: 10% of the benchmark results are better than the evaluated product, and 75% are worse.
• Above average: 25% of the benchmark results are better than the evaluated product, and 50% of the results are worse.
• Below average: 50% of the benchmark results are better than the evaluated product, and 25% of the results are worse.
• Bad: The evaluated product is among the worst 25% of results

## 3 Results

### 3.1 Participant characteristics

From June to August 2024, 30 adolescents were enrolled in the EatsUp® Usability Testing program. The testing requires the participants to use the app for seven consecutive days. The mean ± SD age of participants was 16 ± 0.70 years. 23 (76.7%) were female students, 16 (53.3%) were 16 years old, 30 (100.0%) were students from schools based in Jakarta, and 27 (90.0%) reported that they had used the app for ≥7 consecutive days (Table 3).

**Table 3:** Demographic characteristics of the participant.

Characteristics	Values
Gender, *n* (%)	
Female	23 (76.7%)
Male	7 (23.3%)
Age, *n* (%)	
Age (years, mean ± SD)	16 ± 0.70
15	3 (10.0%)
16	16 (53.3%)
17	10 (33.3%)
18	1 (3.3%)
School Location, *n* (%)	
Jakarta	30 (100.0%)
Duration of EatsUp® app Usage	
<7 days	3 (10.0%)
≥7 days	27 (90.0%)

### 3.2 User experience with the app

**Figure 2:**
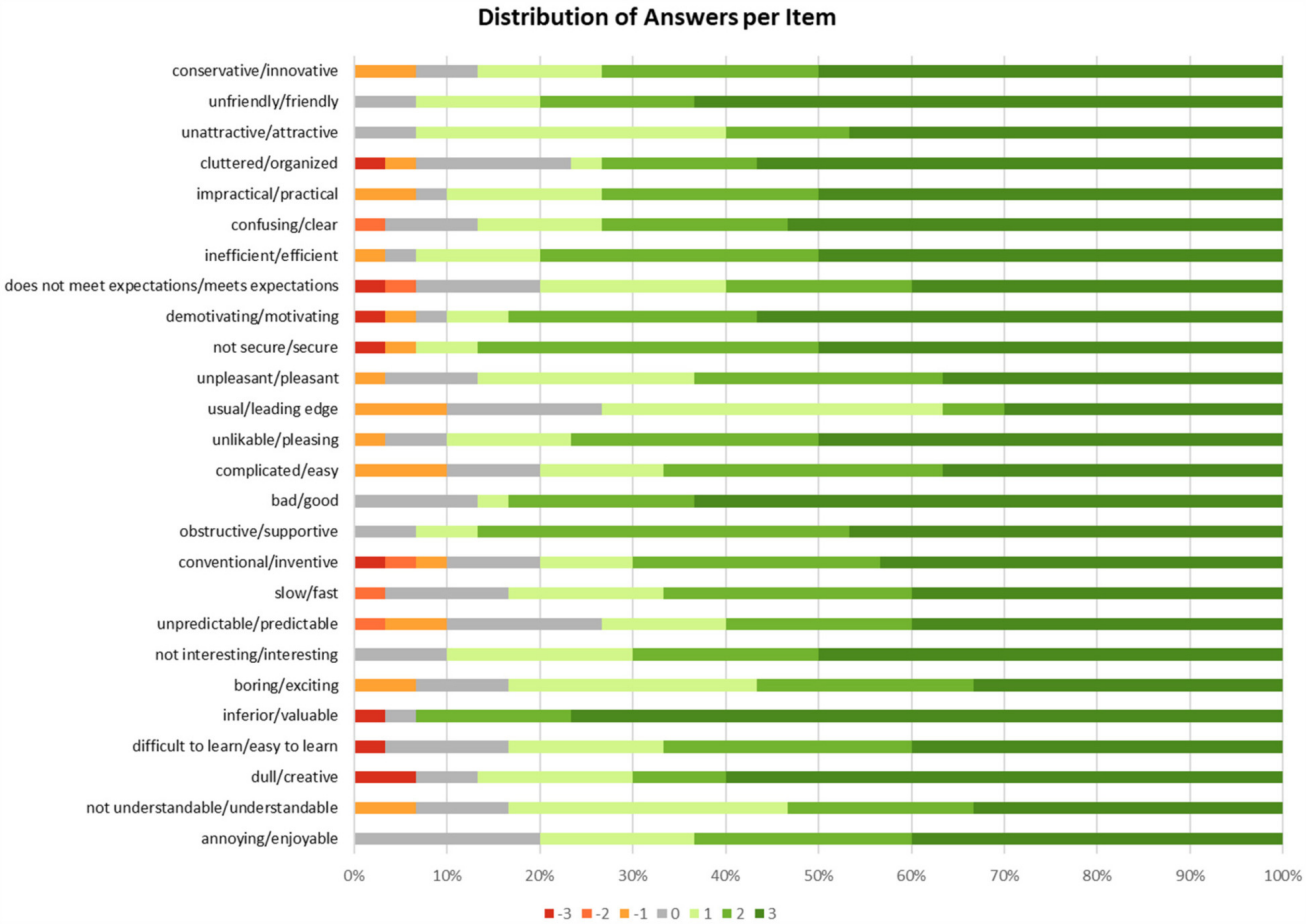
Distribution of answers per item from 30 participants.

The overall results of the EatsUp® UEQ scale were evaluated by examining the mean values for each parameter, based on the UEQ scale ranging from −3 to +3 (7-point Likert scale). Each participant responded to 26 items from the UEQ questionnaire, with the distribution of their response scores for each item presented in Figure 2.

Subsequently, several items were grouped into their respective parameters, as outlined in Table 1. The mean score for each parameter was then calculated for each participant. The overall mean score for each UEQ parameter was then determined by averaging the individual mean scores across all 30 participants. The mean scores are as follows: Attractiveness 2.08, Perspicuity 1.81, Efficiency 2.01, Dependability 1.92, Stimulation 2.12, and Novelty 1.76 (Table 4).

**Table 4:** Average scores of the UEQ parameters for the EatsUp® mobile application.

Parameter	Mean ± SD	Comparison to benchmark	Interpretation
Attractiveness	2.08 ± 0.93	Excellent	In the range of the 10% best results
Perspicuity	1.81 ± 1.11	Good	In the range of the 25% best results
Efficiency	2.01 ± 1.07	Excellent	In the range of the 10% best results
Dependability	1.92 ± 0.94	Excellent	In the range of the 10% best results
Stimulation	2.12 ± 0.90	Excellent	In the range of the 10% best results
Novelty	1.76 ± 0.80	Excellent	In the range of the 10% best results

**Figure 3:**
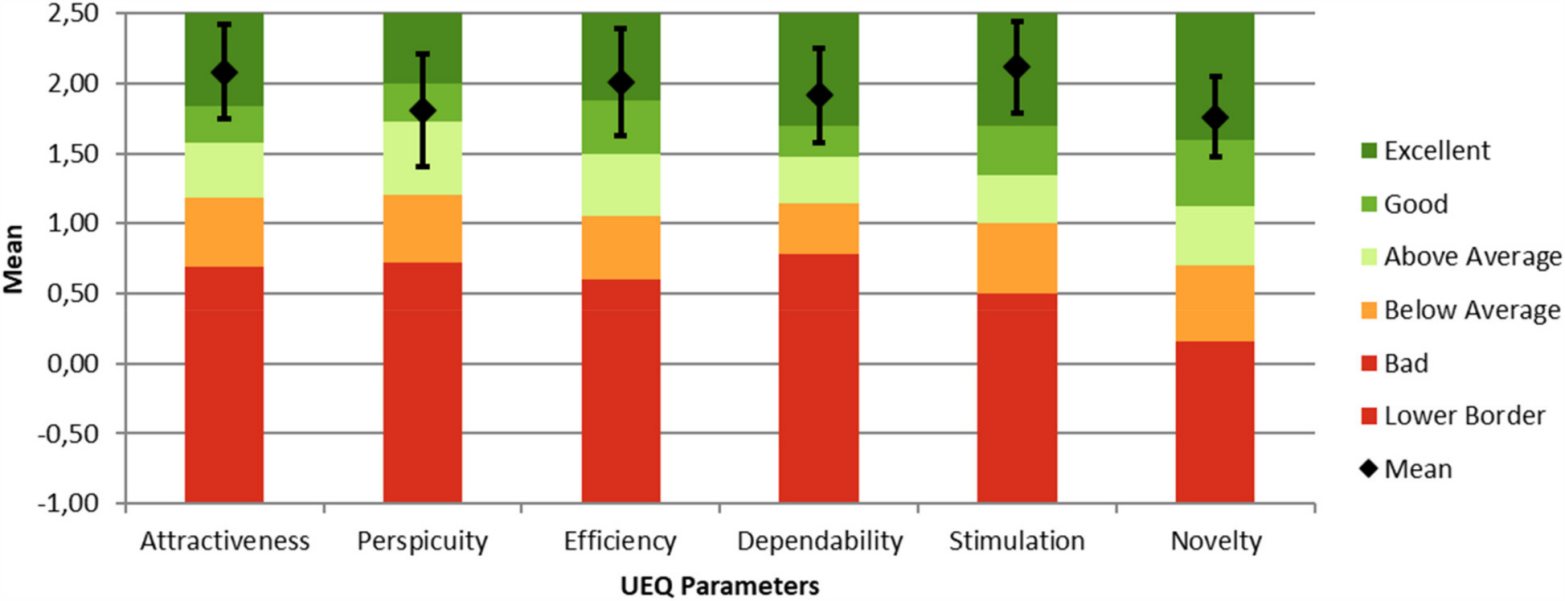
Benchmark diagram of the user experience parameters for the EatsUp mobile application with mean score and 95% CI whiskers.

Figure 3 illustrates that the UEQ results for the EatsUp® application demonstrate a positive user experience, with positive values defined as mean scores greater than 0.8 for each parameter. According to the UEQ benchmark diagram (Figure 3), the EatsUp® application falls into the “Excellent” category for Attractiveness, Efficiency, Dependability, Stimulation, and Novelty, while Perspicuity is rated as “Good”. Thus, based on the UEQ, the overall quality of the EatsUp® application is high, with “Excellent” indicating placement within the top 10% of results and “Good” within the top 25% when compared against the benchmark dataset from previous UEQ studies. This benchmark dataset includes data from 21,175 individuals across 468 studies on various products, updated annually (19).

## 4 Discussion

In this study, usability testing provided valuable insights into the user experience of the EatsUp® application, designed to track dietary intake and physical activity and promote healthy lifestyles among adolescents. The study results show that most participants were female (76.7%), and all participants (100%) were students from schools in Jakarta. Both factors may influence perceptions of app usability. Prior research finds that females often have distinct usability preferences, such as favoring multiple options over linear sequences, employing comprehensive information-processing strategies, and relying on additional navigation cues (31). Additionally, environmental factors such as peer influence, high awareness, and facilitating conditions like ease of access are crucial in shaping user perceptions of mobile app usability (32). The test results indicate that the app provides an overall positive user evaluation with “Excellent” and “Good” ratings across various user experience items, suggesting that this app suits adolescents. This finding aligns with a previous study that emphasizes the importance of usability in the success of health-related mobile apps, particularly those targeting adolescents (12). The EatsUp® application scored “Excellent” in parameters like attractiveness, stimulation, and novelty, which are key factors for sustained engagement among younger users. Moreover, the efficiency and dependability also scored “Excellent”, reflecting the app's reliability and ease of use, which are essential for user acquisition and retention (29).

Attractiveness is evaluated using visually oriented items, such as annoying or enjoyable, and unlikeable or pleasant, while stimulation reflects the emotional response when using the application (see Table 1). Prior research finds that health applications with visually engaging elements such as vibrant colors, animations, and videos can capture users' initial interest (33). Moreover, previous studies have emphasized that an attractive and aesthetically pleasing User Interface (UI) is crucial for adolescents, as it enhances motivation for using the app regularly (34). Previous studies also identified reliability and security influencing users' decisions to download and use apps. Reliability concerns app malfunctions or crashes, while security concerns data protection and potential exposure to fraud (35). This study assessed both aspects under Dependability, which received an excellent rating, indicating strong user confidence in the app's reliability and security. Overall, a mobile health app with high usability will increase user engagement through user interaction with the system. Thus, features promoting usability and engagement can increase the likelihood of successful behavior change (36).

Based on the UEQ benchmark comparison, perspicuity is the only component classified as “good” (top 25%) as compared to the other five parameters that are classified as “excellent” (top 10%), representing a potential area for improvement for the EatsUp® mobile app. Perspicuity, which reflects how easily users can understand and navigate an application, is critical to mobile app usability. Key elements of perspicuity, such as interface clarity, intuitive navigation, and straightforward instructions, are essential in helping users learn and use the app efficiently without unnecessary effort or confusion. This parameter is vital for initial user engagement and sustained application use (37–39). In this study, perspicuity received lower average ratings on “complicated-easy” and “not understandable-understandable”. Improving perspicuity can be achieved by enhancing the UI to ensure that navigation is clear, consistent, simple, and responsive. Implementing these improvements would be a practical approach to improve the perspicuity and ensure users can navigate the app seamlessly (40).

As a mobile application designed to support digital health interventions, EatsUp® requires careful consideration of the Digital Determinants of Health (DDOH) to ensure that this mobile application is not only effective but also equitable across diverse populations. Emphasizing equity in digital health is essential to reduce disparities and promote access among underrepresented groups, helping ensure that digital tools are inclusive by design (41). DDOH includes a range of aspects such as ease of use, usefulness, interactivity, digital literacy, digital accessibility, digital availability, digital affordability, algorithmic basis, technology personalization, and data poverty and information asymmetry (42). In alignment with several of these aspects, the UEQ results for the EatsUp® application reflect strong interactivity and user engagement, with Attractiveness and Stimulation both rated as “Excellent”. The Efficiency score also rated “Excellent” which indicates a high level of ease of use. Additionally, EatsUp® was specifically adapted for adolescent users through feature modifications that enhance its usefulness, and is available on both Android and iOS platform, enhancing its digital accessibility and availability. However, an important area for improvement lies in the Perspicuity, which scored relatively lower compared to other UEQ parameters. This suggests that some users with lower levels of digital literacy may face challenges in understanding and navigating the application. Therefore, it is essential to enhance inclusivity and support broader implementation across diverse adolescent populations, particularly those with limited digital literacy as they are more likely to experience barriers using health mobile applications (43).

Despite the positive findings, there are limitations to this study that should be acknowledged. The predominance of female participants (76.7%). This aligns with previous research that assessed the acceptance of EatsUp®, which also consists of mostly female participants (21). Based on the school location, all participants (100%) were students from schools based in Jakarta. These limit the generalizability of the findings to adolescents as app usability can vary depending on user characteristics and environmental factors. Both parameters can influence the perception of app usability (31, 44). Future studies may include a more diverse sample to capture a broader range of user experiences. Future studies could also explore how different features like gamification can be optimized to increase user engagement and adherence over time.

In conclusion, the 7-day usability testing of EatsUp® suggests that the app provides a positive user experience among adolescents, indicating its potential suitability for this age group. These findings suggest that the app has potential in supporting user engagement, which is a key element in encouraging health-related behavior change. To enhance the generalizability of the results, future studies should include a more diverse sample to reduce bias caused by the large proportion of female participants from schools based in Jakarta. Including more diverse genders, various regions and socioeconomic backgrounds would provide a broader understanding of user needs and experiences. Additionally, future research for EatsUp® should evaluate the effectiveness of EatsUp® as an intervention tool in improving adolescents' adherence to a balanced diet and promoting healthy lifestyle behaviors over a longer duration. It is also recommended to investigate the optimization of aspects such as gamification to increase sustained engagement.

## Data Availability

The datasets presented in this article are not readily available because the dataset contains information that could compromise the privacy of research participants and highly confidential based on ethical committee legacy. Requests to access the datasets should be directed to Erfi Prafiantini, erfi.prafiantini@ui.ac.id.
